# Adnexal Clear Cell Carcinoma Exhibiting Comedonecrosis of the Ear: A Rare Case Treated With Mohs Micrographic Surgery

**DOI:** 10.7759/cureus.65041

**Published:** 2024-07-21

**Authors:** Helen Z Chen, Mohamad Jabin, Michelle Tarbox, Russell Akin, Ashley Sturgeon

**Affiliations:** 1 Dermatology, Texas Tech University Health Sciences Center, Lubbock, USA; 2 Medicine, Texas A&M College of Medicine, Bryan, USA

**Keywords:** invasive clear cell squamous carcinoma, adnexal neoplasm, immunohistochemistry staining, mohs surgery, adnexal tumor

## Abstract

Adnexal clear cell carcinoma exhibiting comedonecrosis (ACCCC) is a rare, cutaneous, malignant neoplasm with limited reported cases since its discovery. ACCCC is characterized by unique clinical and histological features, demanding a precise diagnosis due to its potential for aggressive behavior and early metastases, distinct from other cutaneous tumors with clear cytoplasmic cells. We present the case of an 81-year-old male with a history of multiple non-melanoma skin cancers, who presented with a 5 mm erythematous papule on his left tragus. Initial tangential shave biopsy results were invasive clear cell squamous cell carcinoma that was moderately differentiated. Subsequent Mohs micrographic surgery (MMS) necessitating a full-thickness skin graft reconstruction was performed. Histopathological examination afterward confirmed ACCCC with pleomorphic epithelial cells, clear cytoplasm, and central comedonecrosis. Immunohistochemistry supported adnexal differentiation and squamous features. To our knowledge, this is the fifteenth reported case of ACCCC as well as the first documented case of ACCCC treated with MMS, offering a novel approach to managing this rare malignancy.

## Introduction

Malignant adnexal neoplasms occur infrequently, representing about 1-2% of cutaneous epithelial tumors [1]. These carcinomas often arise in areas with high concentrations of apocrine and eccrine glands like the head, neck, fingers, and toes, although they may rarely present on the trunk or extremities [1]. Adnexal clear cell carcinoma exhibiting comedonecrosis (ACCCC) as an entity was first described by Chaudhry when a group of adnexal tumors with similar histologic findings showed an aggressive clinical outcome compared to their other clear cell cytoplasm counterparts [2]. Since his initial discovery, there have been limited reported cases worldwide. Unlike clear cell variants of squamous cell carcinomas or trichilemmal carcinomas, ACCCC possesses an increased risk of local recurrence, lymph node spread, and distant metastases [2,3]. Careful histologic evaluation and immunohistochemistry are therefore imperative to differentiate ACCCC from more indolent cutaneous tumors containing clear cytoplasmic cells. Histologically, it exhibits a distinctive zonal architecture with peripheral squamoid cells transitioning to clear cells containing intracellular glycogen, accompanied by central comedonecrosis [2]. Immunohistochemistry reveals diffuse epithelial membrane antigen (EMA) and focal carcinoembryonic antigen (CEA) expression in clear cell areas, supporting adnexal differentiation. The management of ACCCC has traditionally involved radical surgery with concurrent regional lymph node dissection when applicable [4]. While wide local resection remains the standard, this case introduces Mohs micrographic surgery as a novel approach, underscoring the need for a comprehensive multi-modal strategy in managing the aggressive nature of ACCCC. Chemotherapy has limited efficacy, emphasizing the importance of innovative surgical interventions. This report contributes to the evolving understanding and potential treatment options for ACCCC, as well as highlighting an important clinical diagnosis that can often be mistaken for more indolent neoplasms like cutaneous squamous cell carcinoma.

## Case presentation

**Figure 1:**
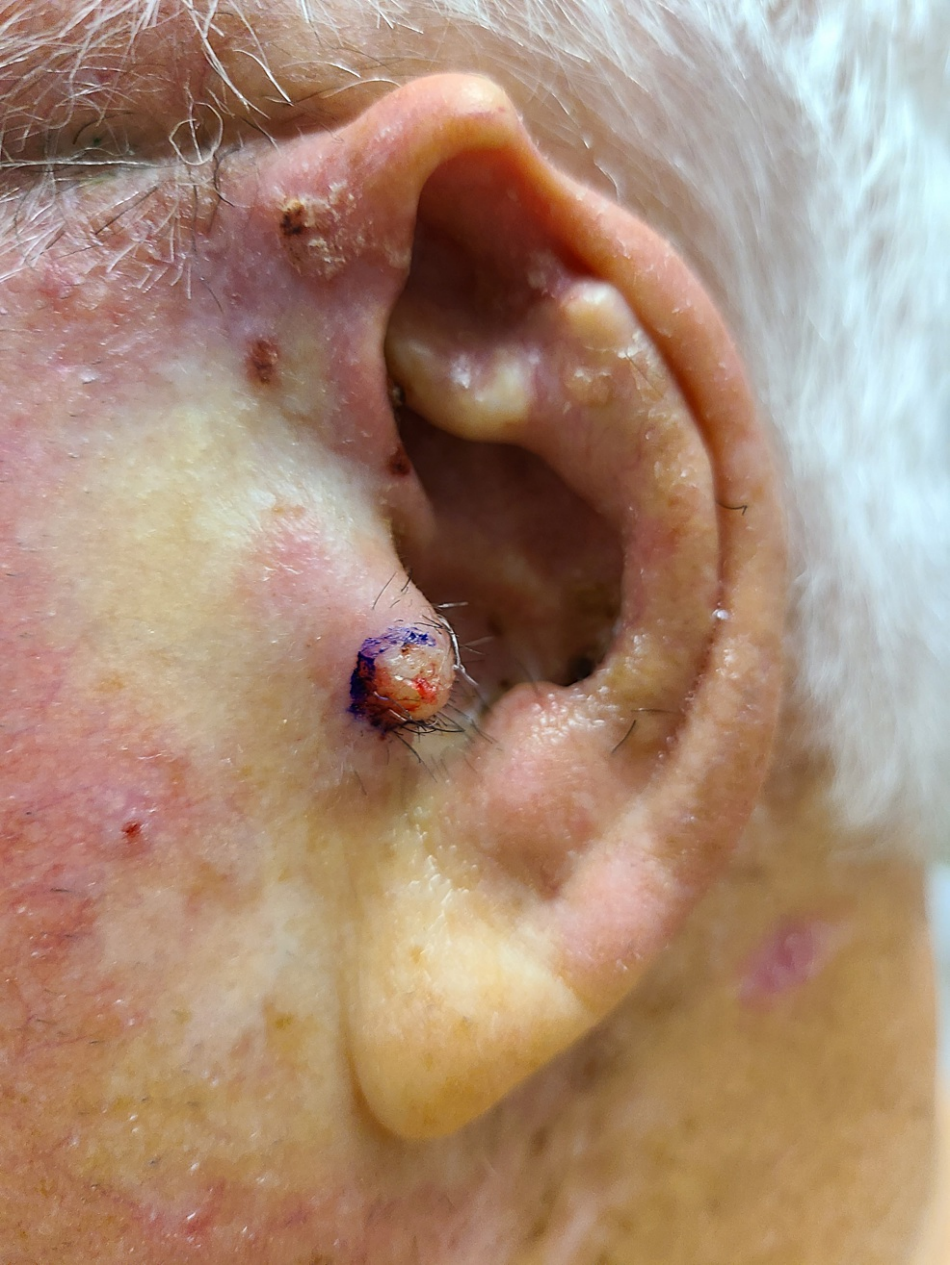
A 5 mm, painful, skin-colored papule on the left tragus before the initial tangential shave biopsy

**Figure 2:**
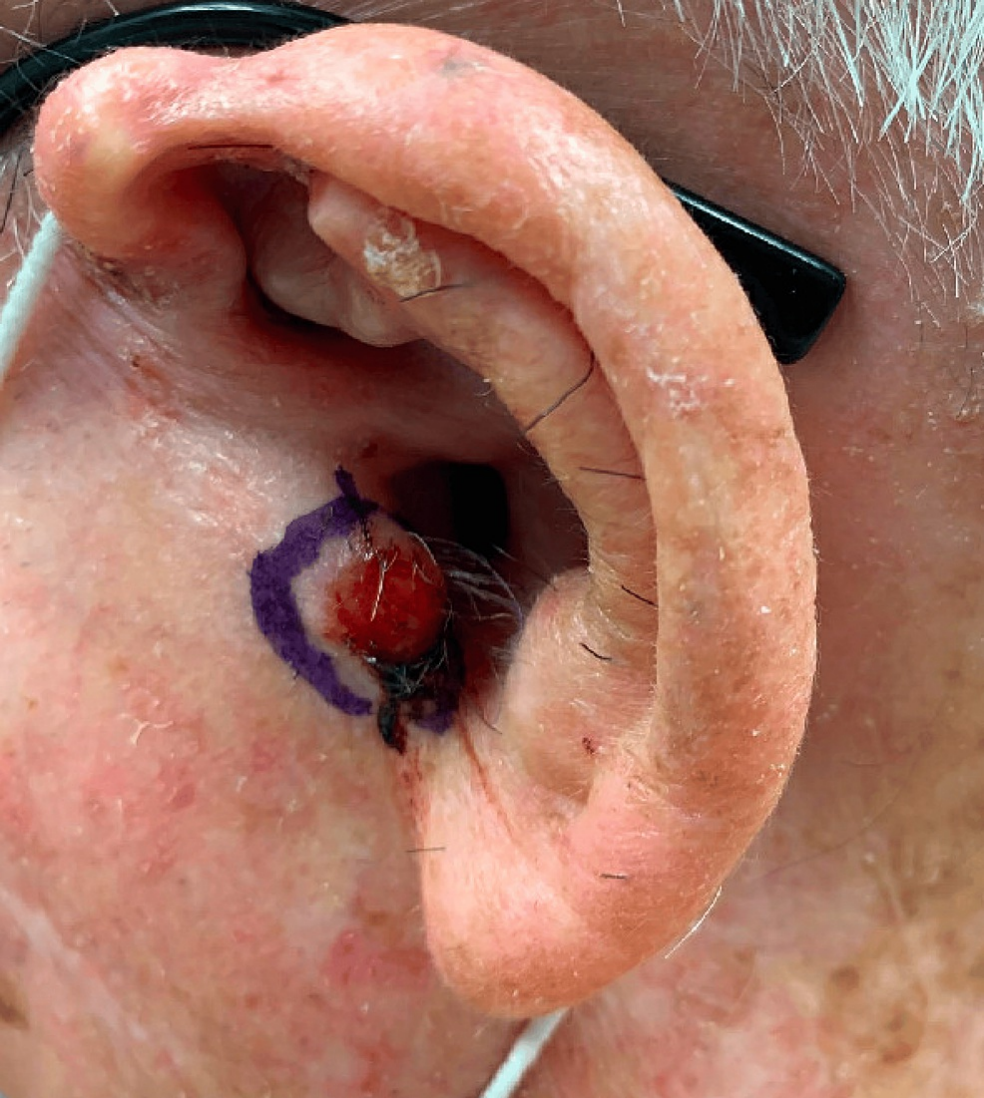
A 17 x 15 mm erythematous nodule on the left tragus before Mohs micrographic surgery

**Figure 3:**
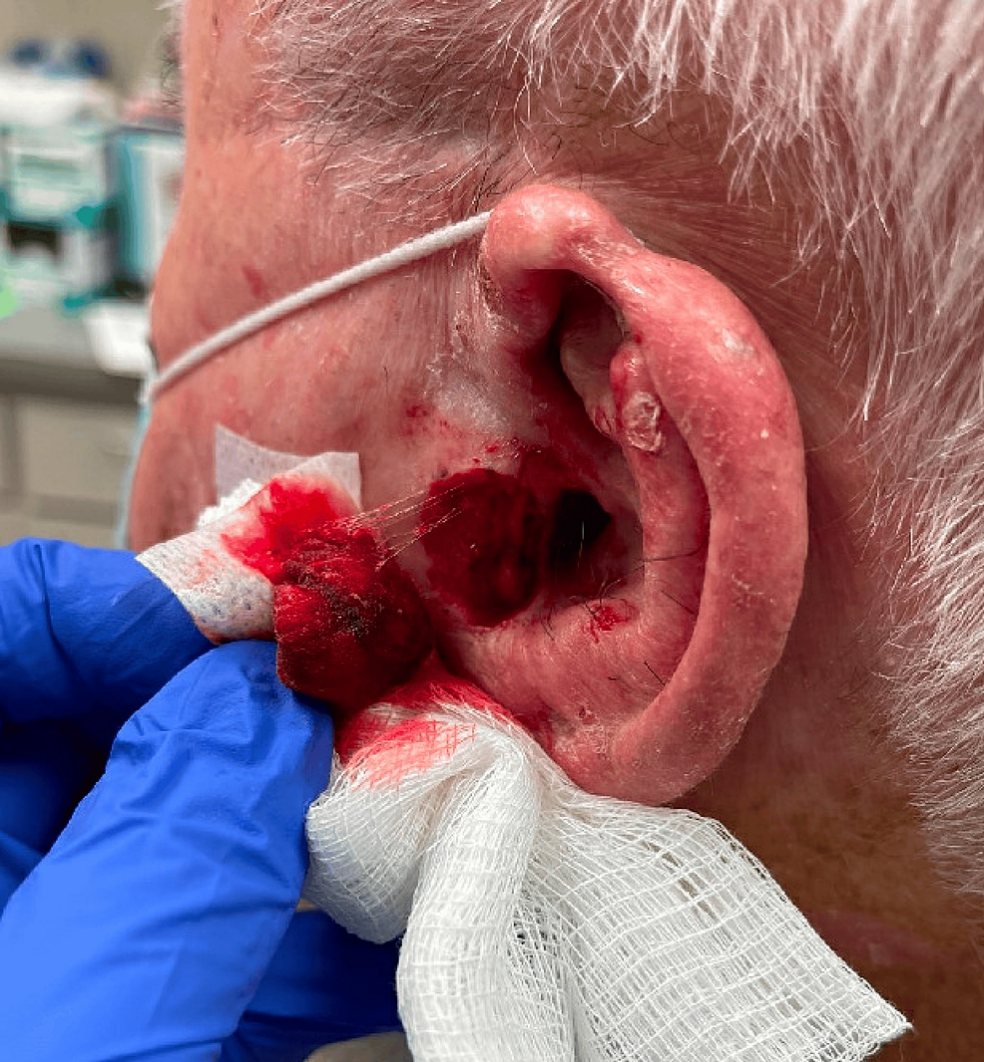
After the first stage of Mohs micrographic surgery, showing a final defect size of 24 x 19 mm extending to subcutaneous tissue and cartilage

**Figure 4:**
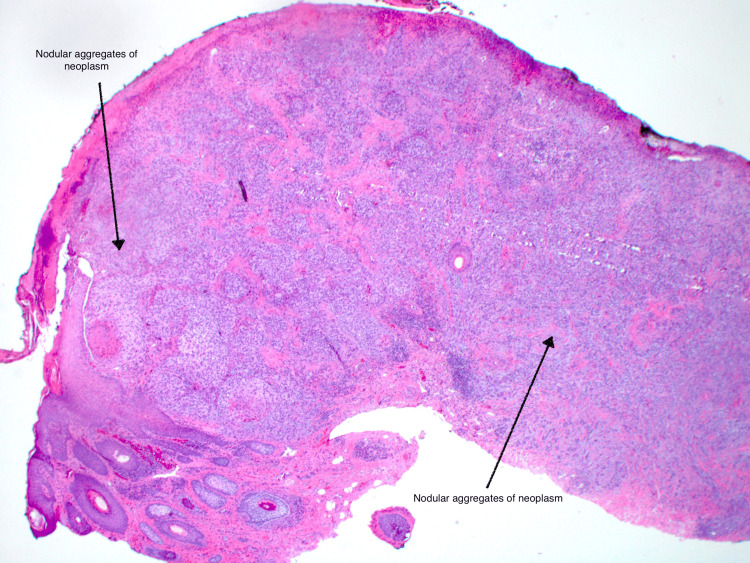
Pathology with H&E stain of the excised specimen showing pleomorphic epithelial cells with focally clear cytoplasm invading through the epidermis in nodular aggregates as well as central areas of comedonecrosis

**Figure 5:**
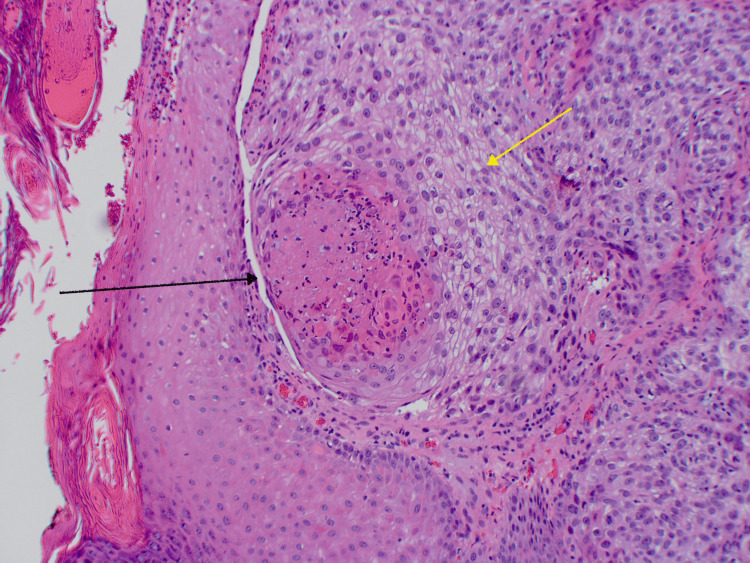
Higher magnification showing peripheral squamous cells transitioning toward clear cells (yellow arrow) with central comedonecrosis (black arrow)

**Figure 6:**
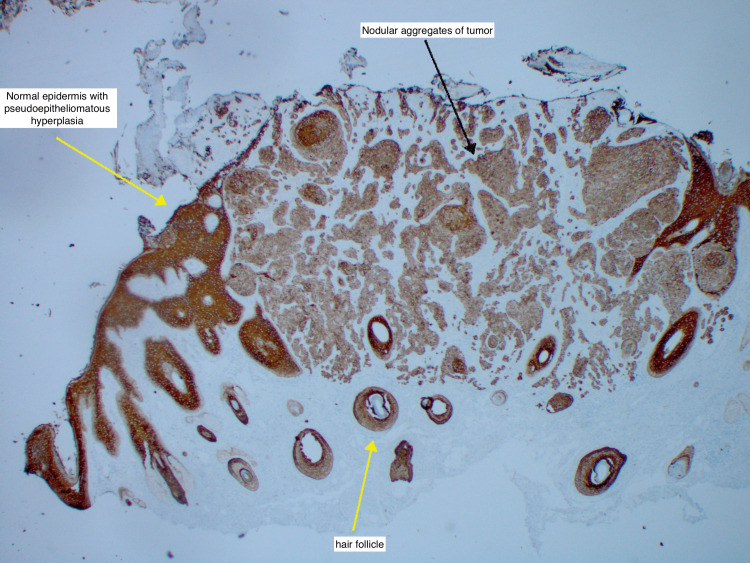
Pancytokeratin stain showing diffuse positivity throughout the tumor nodules (black arrow) The yellow arrow shows a normal hair follicle and pseudoepitheliomatous hyperplasia of the surrounding epithelium.

**Figure 7:**
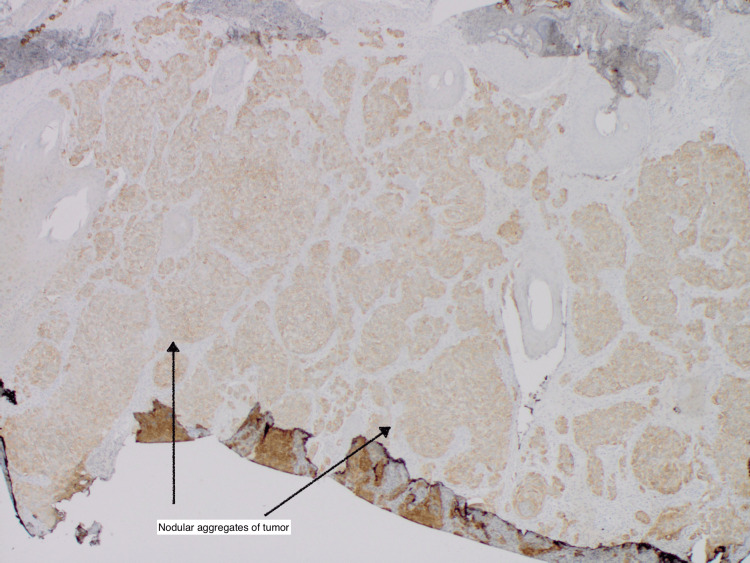
Epithelial membrane antigen (EMA) stain was diffusely positive throughout the tumor (black arrow)

**Figure 8:**
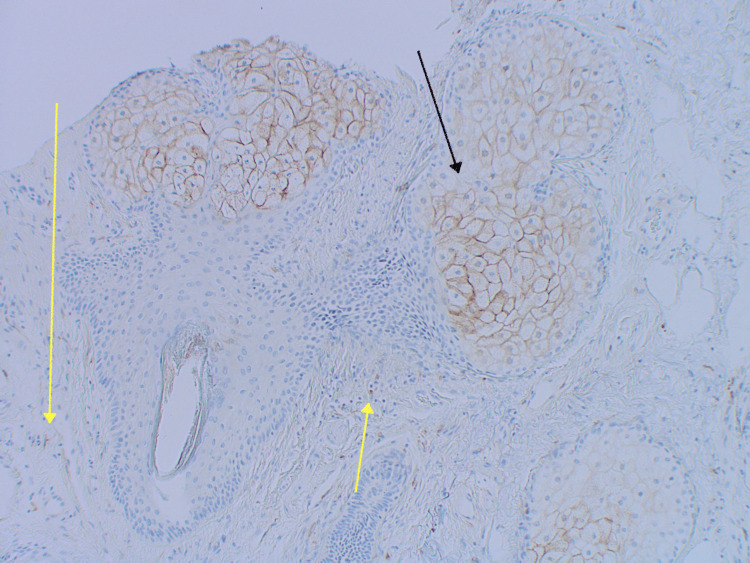
Carcinoembryonic antigen (CEA) showing positive staining of sebaceous glands (black arrow) as well as focal staining with non-ductal involvement throughout the tumor (yellow arrow)

An 81-year-old male with a history of numerous non-melanoma skin cancers (NMSC) presented with a tender 5 mm erythematous papule on his left tragus of unknown duration (Figure 1). Initial tangential shave biopsy revealed invasive clear cell squamous cell carcinoma. On return for MMS, the painful, erythematous lesion had increased in size to 17 x 15 mm ulcerated nodule on his left tragus (Figure 2). After a single stage, clear, deep, and peripheral margins were achieved resulting in a 24 x 19 mm surgical defect extending to the cartilage and subcutaneous tissue (Figure 3). This defect was repaired via a full-thickness skin graft. The pathology of the excised specimen demonstrated a tumor composed of pleomorphic epithelial cells with focally clear cytoplasm invading through the epidermis in nodular aggregates (Figure 4) Central areas of comedonecrosis were identified within the tumor nodules (Figure 5). The immunohistochemical analysis highlighted adnexal differentiation, supported by positive staining for pan-cytokeratin (Figure 6), cytokeratin 17, epithelial membrane antigen (Figure 7), and carcinoembryonic antigen (Figure 8), which is a consistent staining pattern for all cases of ACCCC. Diffuse p63 positivity confirmed squamous differentiation as well. The final diagnosis was an exceptionally rare adnexal clear cell carcinoma with high-risk features of comedonecrosis and deep margin involvement, now cleared with Mohs surgery. Due to the aggressive nature of these tumors, close clinical follow-up is warranted to monitor for recurrence and metastasis.

## Discussion

ACCCC predominantly targets the hair-bearing skin of the head and neck in older individuals, with a median age of 71 years [2]. Lesions manifest as singular ulcerated or crusted papules, nodules, or plaques ranging from 0.5 to 3 cm [5]. At a histological level, ACCCC showcases unique zonal architecture, starting with peripheral squamoid cells and transitioning toward clear cells with notable intracellular glycogen [2]. The hallmark feature includes central comedonecrosis within these nests.

Immunohistochemistry shows the expression of EMA and CEA limited to clear cell areas, supporting adnexal differentiation, likely originating from hair follicles rather than eccrine/apocrine glands [5]. The clear cell alterations arise from the accumulation of intracellular glycogen, distinguishing them from sebaceous differentiation through the absence of cytoplasmic microvacuolization and positive diastase-sensitive periodic acid-Schiff (PAS) staining.

The primary differentials encompass clear cell squamous cell carcinoma (SCC) and tricholemmal carcinoma [6,7]. ACCCC stands apart from clear cell SCC due to its zonal architecture featuring central comedonecrosis, predominant reticular dermal involvement, and adnexal-type immunoexpression of EMA/CEA [2]. Its differentiation from tricholemmal carcinoma lies in the lack of palisading cells or connections to follicular structures [7]. A thorough histologic evaluation is crucial to distinguish ACCCC from other clear cell carcinomas.

Approximately 30% of ACCCC cases experience recurrence and/or metastasis to lymph nodes and lungs [2]. Multiple studies have stressed that radical surgery, involving concurrent regional lymph node dissection when appropriate, remains the gold standard for managing these aggressive tumors [4]. Although wide local excision is the mainstream treatment, the tendency for lymphatic and distant spread warrants an aggressive multi-modal strategy, when feasible, to mitigate the risk of morbidity from uncontrolled disease. Chemotherapy has been used only sporadically for residual or widespread disease [4].

## Conclusions

We present a case of ACCCC, a rare and potentially aggressive neoplasm distinguished by its manifestations of squamous differentiation, clear cell alterations, and the presence of comedo-type necrosis. Previous studies have treated this malignancy with wide local resection, but MMS has not been reported. To our knowledge, this is the first documented case of ACCCC treated with MMS. Since his treatment, the patient has been found to have additional NMSC but has had no evidence of recurrent or metastatic disease of ACCCC at his two-year follow-up. Given the aggressive nature of this disease, accurate diagnosis and close follow-up is imperative.
